# Chikungunya fever: pathogenesis and mechanisms underlying pain symptoms

**DOI:** 10.3389/fimmu.2025.1679385

**Published:** 2025-11-11

**Authors:** Wuping Sun, Shuqi Shi, Shengyun Liao, Mingzhu Zhai

**Affiliations:** 1 Department of Clinical Laboratory Medicine, Fifth Affiliated Hospital, Southern Medical University, Guangzhou, China; 2 Shenzhen Yilifang Biotech CO., LTD., Shenzhen, China; 3 Center for Medical Experiments (CME), Shenzhen Guangming District People’s Hospital, Shenzhen, China

**Keywords:** chikungunya virus, arbovirus, pain, arthralgia, myalgia, inflammation, neuroinvasion, cytokines

## Abstract

Chikungunya fever (CHIKF), caused by the Chikungunya virus (CHIKV) and transmitted by Aedes mosquitoes, has rapidly evolved from localized outbreaks to a significant global health threat. While the initial high fever is often debilitating, it is the severe and frequently long-lasting pain, affecting joints (arthralgia), muscles (myalgia), and sometimes nerves (neuropathic pain), that truly characterizes the disease’s impact on sufferers. This review explores how CHIKV infection triggers both acute pain and persistent chronic pain. We examine the mechanisms by which the virus directly damages tissues, incites extensive inflammation, invades the nervous system, and potentially manipulates the immune response, leading to autoimmune-like attacks. Understanding these processes is essential, as current treatments mainly focus on symptom management, and there are no specific antiviral therapies available. Identifying the factors that contribute to the persistence of pain is critical for developing targeted and more effective therapeutic interventions, ultimately alleviating the long-term burden of this debilitating disease.

## Introduction

Chikungunya fever (CHIKF) is an arboviral disease caused by the chikungunya virus (CHIKV), which belongs to the Togaviridae family and the Alphavirus genus (1). The virus is primarily transmitted by mosquitoes of the *Aedes aegypti* and *Aedes albopictus* species, which are widespread in many regions (2). CHIKV was first isolated in Tanzania in 1952. Initially, the disease was endemic to Africa, Asia, and the Indian subcontinent. However, since 2013, it has rapidly expanded into the Americas, infecting over 1.5 million individuals (3). The adaptation of CHIKV to Aedes albopictus, which is capable of transmitting the virus in temperate climates, has increased the risk of outbreaks in regions previously considered unaffected. The rapid spread of the virus is further driven by increased international travel and trade, raising concerns about its potential to establish new transmission cycles in Europe, the Americas, and other temperate zones (4, 5). The name “chikungunya” is derived from the Makonde language, meaning “that which bends up,” referencing the severe joint pain and postural changes characteristic of the disease.

CHIKF is characterized by acute, often debilitating symptoms, including high fever, severe polyarthralgia (joint pain), myalgia (muscle pain), headache, rash, and nausea (6). A hallmark feature is intense, acute joint pain, typically affecting small joints, and often accompanied by swelling and stiffness. Notably, a significant proportion of patients develop chronic arthralgia that can persist for months or even years, severely impairing daily function and quality of life (7). Factors influencing the severity and duration of symptoms include patient age, pre-existing health conditions, and the initial disease course (8, 9). Older adults and individuals with pre-existing rheumatological conditions are at higher risk of developing persistent symptoms, underscoring the importance of ongoing research to better understand and manage the disease (10). The virus invades human cells by binding to receptors such as MXRA8, rapidly replicates, and causes extensive tissue damage (11). This tropism for musculoskeletal tissues, including myocytes (muscle cells), directly contributes to the characteristic myalgia, while infection of joint tissues underlies the debilitating arthralgia. Currently, there is no specific antiviral treatment for CHIKF. Management remains symptomatic, primarily involving analgesics and anti-inflammatory agents. Recently, cases of CHIKV infection have been identified in the Guangdong province, China, with gradual spread, posing a certain threat to the health of local residents and socioeconomic stability (12, 13). Therefore, a better understanding of the underlying mechanisms of pain in CHIKF could help develop targeted therapies, improving pain management and overall patient outcomes. This review summarizes the occurrence, potential mechanisms, and emerging therapeutic approaches for the pain syndrome, a primary symptom of CHIKF, aiming to provide a reference for clinicians and patients alike.

## Virology and pathogenesis of CHIKV

CHIKV is a positive-sense, single-stranded RNA virus with a genome approximately 12 kilobases in length, encoding four non-structural proteins (nsP1-4) and four structural proteins (C, E1, E2, and 6k) (14). The enveloped virus measures around 60–70 nm in diameter and is sensitive to desiccation and temperatures exceeding 58 °C (15).

The virus gains entry into human cells primarily via specific receptors such as MXRA8, which mediates attachment and internalization (11). Following receptor binding, the virus is endocytosed, and the viral envelope fuses with the endosomal membrane, releasing the genomic RNA into the cytoplasm. The viral RNA serves as mRNA for the translation of the non-structural polyprotein, which is processed into nsP1-4. These proteins form the replication complex, synthesizing a negative-strand RNA intermediate, which in turn templates the production of new genomic RNA and a subgenomic RNA that encodes the structural proteins. New virions assemble in the cytoplasm, are processed through the Golgi apparatus, and are released from the host cell by exocytosis (15).

It shows a strong tropism for musculoskeletal tissues, such as synoviocytes, fibroblasts, and myocytes, as well as neural tissues. Upon initial infection at the bite site, particularly of skin fibroblasts and macrophages, CHIKV begins replicating using the host’s cellular machinery (16). Subsequently, it disseminates through the bloodstream and lymphatic system to other tissues, including joints, muscles, and peripheral nerves. This tissue localization of virus results in cellular damage and triggers immune responses, both of which contribute to the hallmark symptoms of high fever, severe joint pain, and systemic inflammation observed in infected individuals (11, 15, 17).

Clinically, the disease manifests with an acute phase characterized by high fever, headache, and debilitating, bilateral, and symmetrical joint pain involving small joints such as wrists, ankles, and phalanges. The joint pain is often severe enough to limit mobility and may be accompanied by swelling in a significant proportion of cases. While neurological complications like encephalitis and meningitis are less common, they can occur, particularly in vulnerable populations. A distinctive and persistent feature of Chikungunya infection is the progression into a chronic phase, during which joint pain, fatigue, and musculoskeletal dysfunction can persist for months or even years, greatly affecting the patient’s quality of life. This chronicity tends to be associated with risk factors such as older age and pre-existing rheumatological conditions, with women showing a heightened susceptibility to severe or prolonged symptoms (7, 9, 18). Despite extensive research, the underlying mechanisms driving the persistent pain in the chronic phase remain incompletely understood, though immune-mediated processes, particularly the production of pro-inflammatory cytokines, are believed to play a significant role. The pathogenesis involves a complex interplay between the virus, the host immune system, and environmental factors, which together orchestrate both the acute inflammatory response and the sustained pain symptoms that characterize chronic CHIKF.

## Mechanisms of pain syndrome in CHIKF

The mechanisms underlying pain in CHIKF involve a complex interplay of direct tissue damage, immune responses, and neural sensitization (19, 20). The virus induces cytopathic effects in synovial cells, muscle fibers, and endothelial cells, leading to cell injury and death (17, 21). This damage results in the release of damage-associated molecular patterns (DAMPs), which activate immune cells and nociceptive neurons, thereby amplifying pain signaling (22–24). In affected joints, the disruption of normal synovial homeostasis leads to synovitis and cartilage degradation, which are key contributors to joint pain (25). During the acute phase of infection, a robust immune response is triggered, characterized by the release of pro-inflammatory cytokines such as interleukin-6 (IL-6), tumor necrosis factor-alpha (TNF-α), and interleukin-1β (IL-1β) (26). It has also been demonstrated that IL-17 plays as a putative hallmark of intense arthralgia and age-related serum immune mediator networks during acute CHIKF (27). These cytokines, along with DAMPs, provoke an “immune storm” that floods infected tissues with inflammatory mediators (28–30). These substances sensitize and activate nociceptors, the pain-sensing nerve endings, resulting in heightened pain perception (**Table 1**).

**Table 1:** Key mechanisms and mediators contributing to pain in Chikungunya fever.

Phase	Key mechanisms	Key mediators/cellular events	Clinical pain manifestations
Acute	Direct viral cytopathic effect Peripheral sensitization Inflammatory "cytokine storm"	DAMPs (e.g., HMGB1) Pro-inflammatory cytokines (IL-6, IL-1β, TNF-α, IL-17) Chemokines (CCL2, CXCL8) Immune cell infiltration (Macrophages, Neutrophils)	Severe arthralgia and myalgia Hyperalgesia
Chronic	Central sensitization & Neuroplasticity Persistent neuroinflammation Potential viral persistence / Autoimmunity	Upregulation of TRPV1, TLR4 in DRG Glial cell activation in spinal cord Altered synaptic transmission (e.g., NMDA receptor activation)	Persistent arthralgia Hyperalgesia Allodynia

Moreover, inflammatory cytokines and chemokines such as CCL2 and CXCL8 recruit immune cells like macrophages, neutrophils, and lymphocytes into the tissues, where they release additional mediators that perpetuate inflammation and further sensitize nociceptors (31, 32). This creates an environment akin to adding fuel to a fire, intensifying pain sensations. Additionally, inflammatory mediators such as prostaglandins, which are targeted by non-steroidal anti-inflammatory drugs (NSAIDs), and bradykinin contribute to nociceptor sensitization, making even light touch painfully perceptible (33). This ongoing inflammatory response explains the intense pain characteristic of the acute phase of CHIKF (34). Evidence suggests that the virus can invade peripheral nerves, dorsal root ganglia (DRG) (20), and central nervous system structures (35, 36). Such neuroinvasion can disrupt neural function, alter signal transmission, and lead to neuropathic pain (19).

Persistent viral RNA in neural tissues may sustain chronic pain even after the initial infection has resolved (31). In the chronic phase of disease, pain persists and is often associated with upregulation of pain-related receptors on sensory neurons, such as TRPV1 and TLR4 (37–39). These changes induce neuroplasticity within the DRG and spinal cord, leading to remodeling of neural circuits and increased pain sensitivity, phenomena known as central sensitization (15).

## Central sensitization represents a maladaptive state of the CNS

**Figure 1:**
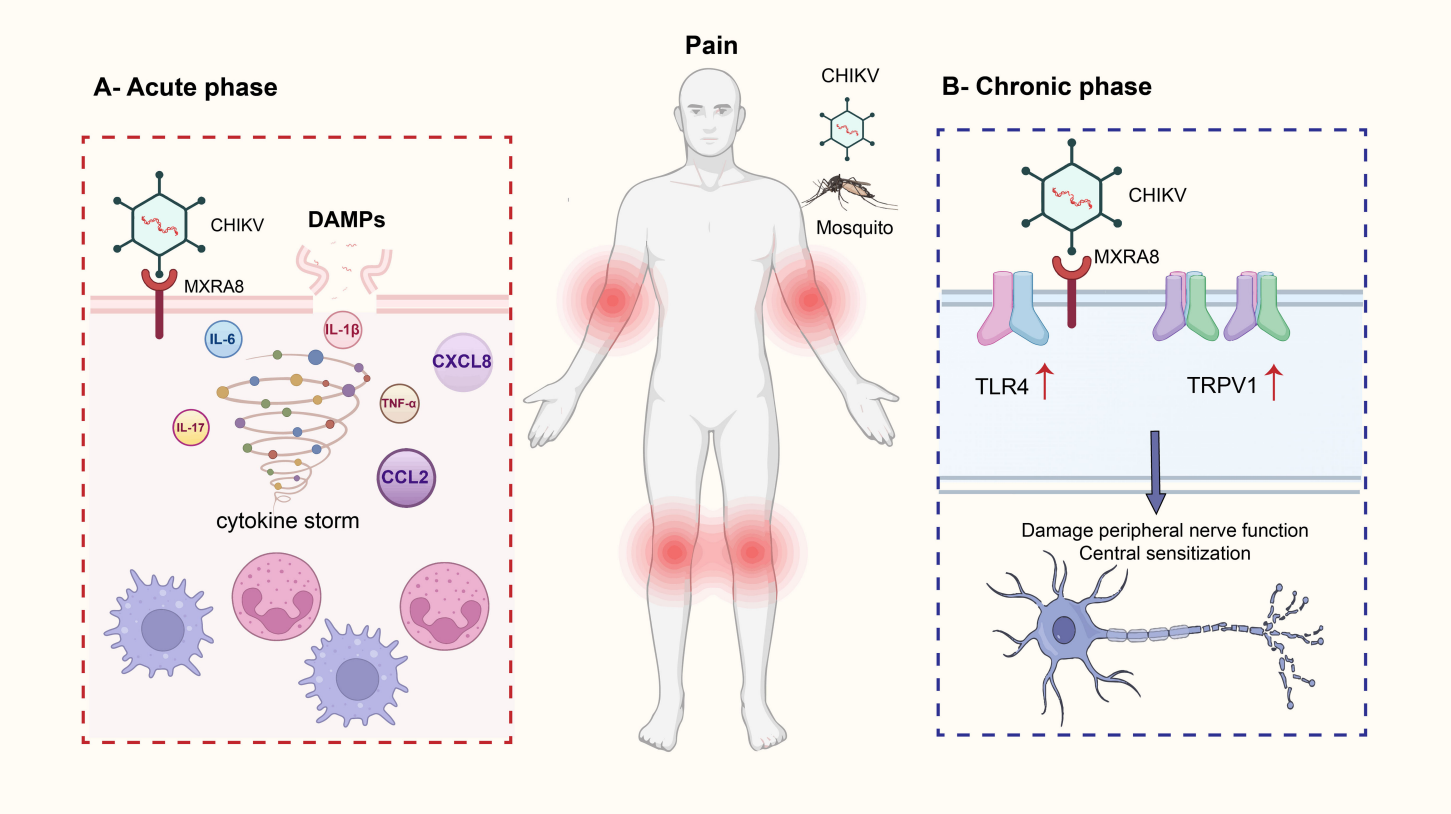
This image illustrates the mechanism by which Chikungunya virus (CHIKV) infection leads to pain. Chikungunya fever is primarily transmitted by the bites of Aedes aegypti and Aedes albopictus mosquitoes. Clinical symptoms typically include high fever, severe polyarthralgia, myalgia, headache, rash, and nausea. In acute phase, CHIKV enters host cells by binding to the MXRA8 receptor, which triggers the release of damage-associated molecular patterns (DAMPs). These DAMPs initiate a cytokine storm, characterized by the release of multiple pro-inflammatory cytokines and chemokines, including IL-6, IL-17, IL-1β, TNF-α, CXCL8, and CCL2. This immune response activates and recruits immune cells such as neutrophils and macrophages, leading to amplified inflammation and acute joint pain. In chronic phase, persistent viral invasion of neural structures results in abnormal nerve function. Chronic pain-related receptors such as TRPV1 and TLR4 become upregulated, promoting neuroplasticity in dorsal root ganglia (DRG) and spinal cord neurons. This contributes to central sensitization, which underlies prolonged and potentially worsening chronic pain.

In the spinal cord, persistent nociceptive input from the periphery leads to increased excitability of dorsal horn neurons. This involves enhanced synaptic efficacy mediated by glutamate receptors (e.g., NMDA receptors), reduced inhibitory control by GABAergic and glycinergic interneurons, and increased activity of glial cells (astrocytes and microglia) which release pro-inflammatory cytokines that further amplify neuronal signaling (24, 38). Concurrently, within the DRG, the cell bodies of sensory neurons undergo transcriptional and translational changes, leading to the increased expression of pain-related channels and receptors (e.g., TRPV1, Nav1.7, TLR4). This neuroplastic remodeling lowers the activation threshold for pain (peripheral sensitization) and amplifies pain signals transmitted to the brain (central sensitization). As a result, the nervous system becomes hyperexcitable, and normal stimuli may evoke exaggerated pain responses (hyperalgesia), while normally non-painful stimuli (like light touch) can become painful (allodynia) (**Table 1**, **Figure 1**).

## Factors influencing pain

While the aforementioned mechanisms directly drive pain, several host-related factors can significantly influence an individual’s susceptibility to and the severity of CHIKF-associated pain. Polymorphisms in genes encoding cytokines or pain-related receptors may affect the body’s response to CHIKV infection. For example, certain gene polymorphisms may lead to excessive production of inflammatory cytokines, increasing the severity of pain and the risk of chronic pain. X-linked polymorphisms in TLR7 and TLR8 genes are associated with protection against Chikungunya fever (40, 41). Elderly patients and those with pre-existing joint disorders are more likely to develop chronic pain after CHIKV infection (7, 9). There is also gender-specific differences in pain perception and immune response. Generally, women may be more sensitive to pain, which may affect the course and severity of CHIKF (42). Taken together, the mechanisms underlying pain in CHIKF encompass direct viral damage, immune-mediated inflammation, neural plasticity, and genetic predispositions. Understanding these interconnected processes is crucial for developing effective therapeutics aimed at reducing both acute and chronic pain associated with the disease.

## Clinical manifestations and challenges in pain management

Chikungunya is a mosquito-borne viral disease that causes acute and chronic pain symptoms, particularly polyarthralgia and myalgia. The pathogenesis begins with the infection of specific cells, which triggers an inflammatory response that underlies the clinical symptoms during the acute phase. In the acute phase, patients typically experience intense inflammatory arthralgia and myalgia, often accompanied by fever and joint swelling (43, 44). The pain develops rapidly and can be severe, significantly impairing mobility and daily functioning. As the disease progresses, some individuals develop persistent arthralgia and neuropathic pain, which are more difficult to treat and can substantially affect quality of life. These prolonged symptoms pose a major management challenge, partly because they often overlap with other arboviral infections such as dengue and Zika virus (45).

Diagnostic challenges exist because the symptoms of CHIKF overlap with those of other arboviral diseases, such as dengue fever and Zika virus infection. Persistent inflammatory joint pain, sometimes evolving into distinct neuropathic pain (burning, tingling). Telling CHIKF pain apart from dengue or Zika early on can be tricky, relying on symptom patterns and lab tests. During the acute phase, RT-PCR can detect viral RNA in blood, while serological assays are valuable for diagnosing past infections or ongoing immune responses during later stages (46–48). The incubation period ranges from 4 to 12 days, with most symptoms resolving within ten days, though joint pain may persist for weeks or months beyond the initial illness. Effective diagnosis in regions where multiple arboviruses are endemic relies on an integrated approach that combines clinical, laboratory, and epidemiological data. Although diagnostic advancements have improved accuracy, differentiating CHIKF remains complex, underscoring the importance of comprehensive testing to guide management.

Currently, the pain management of CHIKF are primarily supportive, aimed at relieving pain, inflammation, and swelling, which mainly relies on non-steroidal anti-inflammatory drugs (NSAIDs), corticosteroids, analgesics (e.g., Acetamino-phen, Opioids) and neuropathic agents (e.g., Gabapentin, Amitriptyline). However, the effect of these drugs is limited, and long-term use may have adverse effects (49). Emerging therapies are exploring targeted approaches, such as cytokine inhibitors and TRPV1 antagonists, are being studied, which may provide new ideas for pain treatment. The emerging understanding of mechanisms points to promising future targets: drugs blocking specific cytokines (IL-6, TNF-α) (50), inhibiting overactive pain receptors (TRPV1 antagonists, TLR4 antagonists) (24, 38), or modulating the immune response driving chronicity, offering potential for more precise pain control. Despite ongoing research, there are no specific antiviral treatments for CHIKF. Supportive care remains the mainstay, emphasizing symptomatic relief. Although agents like ribavirin have been investigated, their efficacy is uncertain. Severe neurological or ocular complications may necessitate hospitalization. Patients are advised to rest, stay well-hydrated, and avoid medications like aspirin that increase bleeding risk. Prevention primarily relies on vector control and reducing mosquito exposure (51). Public health measures include the use of insect repellents, protective clothing, elimination of breeding sites, and insecticide-treated nets. Several vaccine candidates are currently in clinical trials, aiming to develop a safe and effective vaccine that offers long-term protection and reduces disease burden. Overall, while research continues, effective antiviral therapies and universally protective vaccines remain experimental. In the meantime, comprehensive supportive care, accurate diagnosis, and preventive measures are essential for managing CHIKF.

## Future directions and research gaps

Although significant progress has been made in understanding the pain mechanisms of CHIKF, many questions remain unanswered. For instance, the molecular basis of CHIKV persistence within tissues, particularly in synovial and neural tissues, is not yet fully understood. Moreover, it is unclear what factors drive the transition from acute to chronic pain, whether it is related to viral RNA persistence, immune memory, or neural scarring. Unraveling these mechanisms is crucial for developing targeted interventions. Current animal models have limitations in accurately replicating human pain phenotypes, especially the complex, long-term aspects of CHIKF-associated pain. Developing more precise translational models is essential for advancing our understanding of the underlying mechanisms and for evaluating the efficacy of novel therapies. Creating models that closely mimic chronic arthralgia and neuropathic pain seen in humans would provide valuable insights.

The development of virus-specific antivirals represents an important future direction, as such treatments could prevent the source of pain altogether. In addition, targeted therapies for chronic pain, such as drugs that inhibit neuroinflammation or restore neural homeostasis, warrant further investigation. Improving our understanding of these pathways could offer new avenues for effective pain management in long-term patients. Future research should prioritize interdisciplinary approaches that integrate clinical, epidemiological, and basic biomedical investigations. Such collaboration can deepen our understanding of virus transmission dynamics and the long-term effect of CHIKF. The development of advanced diagnostic tools and innovative therapies, such as targeted antivirals and immunomodulators, has the potential to significantly improve patient outcomes. Overall, addressing these knowledge gaps will be key to advancing the prevention, diagnosis, and treatment of CHIKF and its associated pain.

## Conclusion

CHIKF is a significant emerging infectious disease characterized by debilitating pain that can persist long after the initial infection. Its pathogenesis involves a complex interplay of direct viral tissue damage, extensive inflammatory responses, neural invasion, and host-specific factors. Understanding these mechanisms is crucial for developing targeted and effective therapies. Future research should focus on elucidating the molecular and cellular pathways involved, identifying novel therapeutic targets, and improving animal models to enhance the translation of research findings into clinical practice. Ultimately, effective management of chikungunya-associated pain, both during the acute and chronic phases, requires moving beyond symptomatic treatment. Strategies that target viral replication, modulate immune responses, and restore neural function hold promise for reducing long-term disease burden. Gaining deeper insights into these processes offers hope for lessening the long-term health impacts of this increasingly global threat.
